# Anosognosia in Alzheimer's disease: A neuropsychological approach

**DOI:** 10.1590/S1980-57642008DN10100013

**Published:** 2007

**Authors:** Bárbara Bomfim Caiado de Castro Zilli, Benito Pereira Damasceno

**Affiliations:** 1 Graduate Student, Department of Neurology,Medical School, State University of Campinas (UNICAMP), Brazil.; 2 Professor, Department of Neurology,Medical School, State University of Campinas (UNICAMP), Brazil.

**Keywords:** Alzheimers disease, dementia, anosognosia, agnosia, awareness, doença de Alzheimer, demência, anasognosia, agnosia, consciência

## Abstract

Anosognosia is often found in Alzheimer's disease (AD), but its relationship with cognitivebehavioral changes is not well established.

**Objective:**

To verify if anosognosia is related to cognitive-behavioral disturbances, and to regional brain dysfunction as evaluated by neuroimaging.

**Methods:**

We included AD patients with Mini-Mental State Examination (MMSE) scores of 12 through 24, and Clinical Dementia Rating (CDR) scores of 1 or 2. Dementia diagnosis was based on DSM-IV and NINCDS-ADRDA criteria.We used Self-Consciousness Questionnaire (SCQ) and Denial of Illness Scale (DIS), and following neuropsychological counterproofs: WAIS-R digit span, Rey auditory verbal learning, verbal fluency test (category: animals), Cummings' neuropsychiatric inventory (NPI) and Cornell scale for depression in dementia (CSDD).

**Results:**

We studied 21 patients (12 men, 9 women) with AD (14 mild, 7 moderate), age 72.4±8.5 years, education 4.9± 4.2 years, and MMSE score 18.2±5. SCQ and DIS did not correlate to age, education, or regional cerebral perfusion defects, but they tended to correlate to disease duration (and only SCQ also to MMSE). SCQ and DIS were correlated neither to CSDD, NPI, CDR, nor to any neuropsychological test. Significant correlations were found between SCQ and DIS, as well as between SCQ domain of “moral judgment” and MMSE.

**Conclusion:**

SCQ and DIS were not correlated to age, education, disease duration, cognitive-behavioral measures, dementia severity, or regional cerebral perfusion defects, but were correlated to each other, suggesting SCQ and DIS evaluate similar mental functions.

## Abstract (pt)

Anosognosia é freqüentemente encontrada na doença de Alzheimer (DA), porém sua relação com alterações cognitivo-comportamentais não está bem estabelecida.

**Objetivo:**

Verificar correlações entre anosognosia, alterações cognitivo-comportamentais e disfunções encontradas na neuroimagem (SPECT) da perfusão cerebral.

**Métodos:**

Incluimos pacientes com DA apresentando escores de 12 a 24 no Mini-Exame Mental (MEM) e 1 ou 2 na escala CDR (Clinical Dementia Rating). O diagnóstico de demência baseou-se nos critérios DSM-IV e NINCDS-ADRDA. Usamos o Questionário de Auto-Consciência (SCQ), a Escala de Negação de Doença (DIS); e contra-provas: span numérico do WAIS-R, aprendizado auditivo-verbal de Rey, fluência verbal (categoria: animais), inventário neuropsiquiátrico de Cummings (NPI) e a escala Cornell para depressão na demência (CSDD).

**Resultados:**

Estudamos 21 pacientes (12 homens, 9 mulheres) com DA (14 leve, 7 moderada), idade de 72,4±8,5 anos, educação 4,9±4,2 anos e escore do MEM 18,2 ± 5. SCQ e DIS não relacionaram-se com idade, educação, defeito perfusional cerebral, CSDD, NPI, CDR ou testes neuropsicológicos, mas tenderam a relacionar-se com duração da doença (e apenas o SCQ também com o MEM). Correlações significativas foram encontradas entre o SCQ e a DIS, bem como entre o domínio de “julgamento moral” do SCQ e o MEM.

**Conclusão:**

O SCQ e a DIS não se correlacionaram com idade, educação, duração da doença, alterações cognitivo- comportamentais, gravidade da demência ou defeito regional da perfusão cerebral, mas tenderam a correlacionar- se entre si, sugerindo que SCQ e DIS avaliam funções mentais similares.

Anosognosia (Gk. “gnosis”, knowledge; “nosos”, disease) has been defined broadly as “apparent unawareness, misinterpretation, or explicit denial of illness”^1^, or as impaired insight for behavioral and cognitive problems. Other authors^2^ have approached anosognosia as an impairment of self-consciousness (SC). SC is then conceived as the process by which the subject becomes the object of their own awareness by realizing that they are perceiving something (their own body or the outside world) or reflecting on their own history (autobiography) or projects^2,3^. SC is thus a complex mental function dependent on memory and other cognitive functions, comprising different aspects or degrees, which have to be taken into account in evaluation^2^.

Anosognosia has often been reported in Alzheimer's disease (AD), with a prevalence ranging between 15% and 25%^4,5^. AD involves pervasive changes of attention, perception, memory, humor, and personality, and whose relation with anosognosia is not well understood.

Even the relationship between anosognosia and dementia severity has been inconsistent, partly because other confounding variables (e.g., depression) have not been taken into account. Smith et al.^6^ have found a positive correlation between dementia severity and degree of anosognosia after controlling for depressive symptoms, while other authors^7^ have found similar correlation even without controlling for these symptoms.

As regards the role of depression, various authors^5,6,8-10^ have found it to be negatively correlated to anosognosia in AD patients, while others have not^11^. Migliorelli et al.^9^ found that patients with depressive symptoms (dysthymia) had lower anosognosia scores (i.e., greater insight) than patients with AD who had either major depression or no depression. Therefore, it is important to distinguish between major depression and depressive symptoms when analyzing anosognosia^13^.

The relationship between anosognosia and regional brain dysfunction is not well established. Anosognosia has been associated to right-hemisphere lesions involving the parietal and temporal lobes, thalamus, and basal ganglia^13,14^, as well as the frontal lobes^15-17^. According to Starkstein & Robinson^18^ in a study of stroke patients, the presence of neglect and frontal-subcortical dysfunction may constitute important predisposing factors. Furthermore, Reed et al.^4^ and Starkstein et al.^19^ have found anosognosia associated with a deficiency in the perfusion of the dorsolateral portion of the right frontal lobe. Thus, Lopez et al.^15^ and Stuss & Benson^20^ suggest that the frontal lobes play a relevant role in self-consciousness and monitoring of cognitive tasks. As such, anosognosia would result from a deficit in auto-monitoring due to frontal lobe dysfunction. However, Starkstein et al.^12^ detected no significant difference between AD patients with and without anosognosia, as regards their performance in tests of executive function.

The aim of this study was to verify relationships between anosognosia and cognitive deficits, depressive symptoms, behavioral disturbances, and regional cerebral blood flow in patients with mild to moderate AD. Our hypotheses were that anosognosia would be more frequent and severe (1) the longer the duration of the disease, (2) the more widespread the brain lesions, (3) the more severe the cognitive deficits and dementia, and (4) the lower the depressive scores.

## Methods

We included patients with probable AD consecutively attended at our university hospital, aged 45 to 95 years, and presenting scores from 12 to 24 on Mini-Mental State Examination (MMSE)^21,22^, and scores 1 or 2 on Clinical Dementia Rating (CDR)^23^. Exclusion criteria were any clinically significant cardiac, pulmonary, hepatic or renal disease, chronic exposition to neurotoxic compounds, or previous head trauma with loss of consciousness.Major depressive disorder as a cause of dementia syndrome was excluded when not fulfilling DSM-IV criteria^24^ for depression or when cognitive complaints had not improved after satisfactory treatment of depressive symptoms. All patients gave their informed consent to participate, in accordance with the rules of our Medical School Ethics Committee.

All patients provided a medical history, and underwent physical, neurological, and neuropsychological examination. Diagnosis of dementia was based on DSMIV criteria^24^, as well as on NINCDS-ADRDA^25^ for AD. Computed tomography (CT), magnetic resonance imaging (MRI), cerebral blood flow imaging (SPECT tomography using technetium-99m-HMPAO), electroencephalography, cerebrospinal fluid analysis, and relevant laboratory blood tests were performed to rule out other causes of dementia. SPECT images were interpreted by noting the location, extent and severity of the perfusion defects, and subsequently classified into the following perfusion patterns, according do Holman et al.^26^: A, normal; B, bilateral posterior temporal and/or parietal cortex defects; C, bilateral posterior temporal and/or parietal cortex defects with additional defects; D, unilateral posterior temporal and/or parietal cortex defects with or without additional defects; E, frontal cortex defects only; F, other large (>7 cm) defects; G, multiple small (≤7 cm) cortical defects.

Neuropsychological investigation comprised MMSE, WAIS-R Digit Span for attention^27^, Verbal Fluency test (VF; category: animals' names in one minute) for executive functions^28^, Rey Auditory Verbal Learning test for memory (RAVLT)^28,29^, Neuropsychiatric Inventory (NPI)^30^, and Cornell Scale for Depression in Dementia (CSDD)^31^. CSDD was used to quantify depressive symptoms in our dementia patients.

Assessment of anosognosia was carried out using the Self-Consciousness Questionnaire^2^ and the Denial of Illness Scale^18^ (see Appendix). The Self-Consciousness Questionnaire (SCQ) has fourteen questions, four of them concerning “identity” (Nos. 1, 5, 6, 7), three “knowledge of cognitive disturbances” (metamemory or metacognition: Nos. 2, 3, 4), one “self-evaluation of the affective state” (No. 8), two “knowledge about representation of the body” (Nos. 9, 10), one on “anticipation” (prospective memory: No. 11), one “capacities for introspection” (No. 12), and two “moral judgment” (Nos. 13, 14). The answers were classified as relevant or correct (two points), incorrect (no points), or partly correct (one point). The higher the score, the greater the degree of self-consciousness. The score obtained for each of these aspects of consciousness was divided by the number of questions corresponding to each aspect, giving a total maximum score of 14 points. The answers were checked with the patient's caregiver. The Denial of Illness Scale (DIS) consists of ten items judged by the examiner and used to classify the patients into those with mild, moderate, or severe anosognosia. In DIS, the higher the score (0, 1 or 2) on each item, the greater the degree of anosognosia. SCQ and DIS were translated to Portuguese by the authors, since these scales had not been published or validated in Brazil.

Data analysis by means of Statistica software 6.0 (StatSoft Inc., 2001) used contingency tables (chi-square) and Pearson coefficient for correlations between anosognosia items and neuropsychological tests (counterproofs). The significance level was 5% (two-tailed).

## Results

We studied 21 patients (12 men, 9 women) with probable AD (14 mild and 7 moderate), age 72.4±8.5 years (mean±standard deviation), education 4.9±4.2 years, and score on MMSE 18.2±5.0(Table 1). CT was performed in all patients and MRI in 12. SPECT could not be performed in 5 patients because of operational difficulties or patient refusal. SCQ scores were not related with age, education, or regional perfusion defects found on SPECT images, but tended to correlate with disease duration and MMSE scores, though did not reach statistical significance (r= –0.324 and r=0.317, respectively). In the analysis of correlation between SCQ scores and SPECT patterns (A, B, C, D, E, G; see Table 2), SCQ scores were classified into two subgroups: from 7 to 10.99, and from 11 to 14, since the minimum score was 7.75 and the maximum score, 14. Likewise, DIS scores did not show any correlation to age, education, MMSE, or SPECT data, but tended to correlate to disease duration (r=0.3208), though did not reach statistic significance.

**Table 1:** Demographics and results of Mini-Mental State Examination, Self-Consciousness Questionnaire, Denial of Illness Scale, and cognitive-behavioral evaluation.

Subjects	Sex	Age	Educ	Durat	MMSE	SCQ	DIS	RAVLT	CDR	Atten	VF	NPI	CSDD
AFS	F	83	4	5	14	13.16	8	3.1	1	3	7	0	0
EM	M	77	4	4	25	13.00	4	4.0	1	4	16	0	0
MMK	F	74	1	10	13	12.66	4	2.8	2	3	4	3	1
PF	M	68	4	11	8	12.50	1	0.8	2	2	5	0	0
MAL	F	75	2	9	15	9.00	8	2.1	2	3	7	2	1
ACV	F	62	4	3	12	13.00	2	1.6	2	3	6	0	1
APP	F	74	4	2	22	12.50	4	5.4	1	6	12	3	1
IAR	F	63	4	7	14	7.75	5	2.8	2	3	6	5	1
LB	M	76	1	2	26	13.00	4	6.2	1	5	14	0	0
IM	M	78	2	5	16	11.73	6	3.2	1	7	11	4	2
ADC	F	77	1	4	14	11.83	6	ND	1	ND	ND	ND	ND
OC	M	56	4	8	27	14.00	7	7.2	1	4	13	0	0
FV	M	78	2	2	19	14.00	3	3.8	1	4	6	0	0
TLFM	F	80	8	7	24	13.26	7	4.7	1	7	12	0	0
JLC	M	66	15	3	20	10.50	7	ND	1	ND	ND	ND	ND
PG	M	74	4	8	17	12.08	7	ND	2	ND	ND	ND	ND
PM	M	75	1	2	18	12.50	3	3.0	1	5	9	0	1
EG	M	52	15	3	19	14.00	3	3.1	2	5	8	16	3
MCG	F	85	8	6	20	9.50	8	3.9	1	4	7	0	0
SL	M	78	11	1	23	14.00	1	3.8	1	4	8	1	1
JR	M	69	4	5	16	12.66	5	ND	1	ND	ND	ND	ND

Educ, education; years durat; disease duration; years atten; attention test; SCQ, Self Consciousness Questionnaire; DIS, Denial of Illness Scale; VF, verbal fluency; F, female; M, male; ND, not done; remaining notations, see text (Methods).

**Table 2:** Results of structural (CT, MR) and functional (SPECT) neuroimaging.

Subjects	CT or MRI	SPECT [Holman´s pattern]
AFS	Mild CSCA + HSFSCWM	Mild inferior bifrontal HP [E]
EM	Marked CSCA + HSFSCWM	Moderate diffuse cortical HP [G]
MMK	Moderate CSCA	Normal [A]
PF	Moderate bilateral F-T-P atrophy	Marked bi-T-P-O HP [B]
MAL	Moderate biparietal atrophy	Normal [A]
ACV	Mild CSCA + HSFSCWM	Moderate bilateral T-P-O HP [B]
APP	Normal	ND
IAR	Normal	Normal [A]
LB	Mild CSCA	Normal [A]
IM	Moderate CSCA + HSFCSWM	Multiple bilateral cortical HP areas [G]
ADC	Moderate CSCA + HSFCSWM	Moderate biparietal HP [B]
OC	Normal	Mild bilateral T-P HP [B]
FV	Moderate CSCA + HSFCSWM	Normal [A]
TLFM	Mild CSCA (mostly F)	Mild bilateral T-P-O-cingulate HP [C]
JLC	Mild CSCA (mostly P) + HSFCSWM	Mild bilateral P-O HP [B]
PG	Mild CSCA	Mild bitemporal HP [B]
PM	Mild CSCA	ND
EG	Mild P and medial T atrophy	ND
MCG	Moderate CSCA	Moderate left T and diffuse cortical HP [D]
SL	Mild CSCA	ND
JR	Mild CSCA	ND

CSCA, cortico-subcortical atrophy; HSFSCWM, high signal foci on subcortical white matter; HP, hypoperfusion; F, frontal; T, temporal; P, parietal; O, occipital; ND, not done; Holman´s patterns A, B, C, D, E, G, see text (Methods).

SCQ and DIS scores did not correlate with mood state (CSDD), behavioral changes (NPI), dementia severity (CDR 1 and 2), nor with any of the neuropsychological tests (digit span, verbal fluency, Rey verbal learning). Also, there were no correlations between the different aspects of self-consciousness and any of these cognitive and behavioral variables. The only significant correlations were found between SCQ and DIS scores (r= –0.4482, t=2.185, df=19, p<0.05) and between the domain of “moral judgment” of SCQ and MMSE (r=0.4629, t= 2.2763, df=19, p<0.05).

## Discussion

In agreement with other authors, wee have found no correlations between SCQ or DIS scores and age, education, and disease duration^2^. However, in contrast with these authors, we found no correlation either between SCQ or DIS, and dementia severity as measured by CDR or MMSE scores. As expected, there was a negative correlation between the scores of SCQ and those of DIS, suggesting they measure similar mental changes.

The analysis of each aspect of self-consciousness relative to the neuropsychological (MMSE, verbal learning, digit span, verbal fluency) or neuropsychiatric variables (CSDD, NPI) showed significant positive correlation only between the domain of “moral judgment” and MMSE, in agreement with Gil et al.^2^. This finding is difficult to interpret. It may simply be a statistical artefact without clinical meaning, since changes of moral judgment are usually related to frontal-orbital regions, and only one of our patients had bifrontal hypoperfusion, but without MRI signs of frontal atrophy. Most of our patients had atrophy and hypoperfusion in posterior regions (temporal, parietal, occipital), often bilaterally. Recent studies^32^ have shown moral judgment ability to be a complex function, which requires a whole neurofunctional network, including posterior associative brain regions and integrating semantic-cultural knowledge and appropriate motivational and affective states.

We hypothesized that anosognosia would be worse the longer the disease duration, the more widespread the brain lesions, the more severe the dementia, and the lower the depressive scores. However, none of these hypotheses were confirmed by our findings, probably because our sample size was small and comprised mostly mild dementia cases (67%), without any patients with severe dementia (CDR 3). The lesion model we used (Alzheimer's disease, with degeneration predominantly in temporal-parietal regions) did not allow us to verify the relevant role of frontal dysfunctions in anosognosia, as established by various authors^4,15,16^.Michon et al.^17^ was indeed able to verify that the severity of anosognosia is related to signs of frontal dysfunction but not to the severity of dementia. Thus, in order to tackle these questions, we need to include a greater number of patients with Alzheimer's disease with CDR 1 through 3, to compare to patients with frontotemporal dementia and normal control subjects, while also using neuroimaging methods robust enough to more precisely delimit the brain lesions or dysfunctions.

## Appendix

**Table:**

**A. Self-Consciousness Questionnaire**	8. Do you feel rather happy or unhappy? Why?		
1. What is your name (surname and first name)?	9. Would you say that you are rather fair or dark-haired?		
2. Why have you come to see me?	10. Are you now sitting, standing or lying down?		
3. Do you have any health problems that prevent you from	11. What are you planning to do shortly or tomorrow?		
leading a normal life?	12. If you had to live your life over again, is there anything you		
4. Have you got any problem with your memory?	would like to change? What?		
5. Have you had a job? What was it?	13. Is it a good thing or a bad thing to tell a lie? Why?		
6. What is the first name of your spouse (or partner)?	14. Is it a good thing or a bad thing to give some money or		
7. What is your mother´s first name?	some food to someone who is starving? Why?		

**B. Denial of Illness Scale**			
**Questions**	**Scoring**		
1. Patient minimizes present symptoms (at interview).	0 (no)	1 (once or twice)	2 (more than twice)
2. Patient alludes to there being nothing really wrong with her or	0 (no)	1 (once or twice)	2 (more than twice)
him and that she or he is ready to go home.			
3. Patient (past or present) displaces source of symptoms to organs	(no)	1 (once or twice)	2 (more than twice)
other than brain or complains of symptoms unrelated to the			
central nervous system.			
4. Did the patient at any time admit to fear of death?	0 (yes)	1 (no)	
5. Did the patient at any time admit to fear of invalidism?	0 (yes)	1 (no)	
6. Patient verbally denies being in the hospital.	0 (not at all)	1 (sometimes)	2 (every time)
7. Patient displays, at least on the surface, a carefree, cheerful,	0 (no)	1 (once or twice)	2 (more than twice)
jovial approach to life.			
8. Patient´s behavior during interview is characterized by	0 (no)	1 (once or twice)	2 (more than twice)
nonchalance, coolness, imperturbablity.			
9. Patient displaces fear for his or her own illness to family, older	0 (no)	1 (at least 1 time during	
patients, weaker patients, and so on.		the interview)	
10.Patient projects illness or weakness to family, spouse, and so on	0 (no)	1 (at least 1 time during the interview)	

## Appendix

**Table:**

**Questionário de Auto-consciência (Gil et al., 2001)**	8. Você se sente feliz ou triste? Por quê?		
1. Qual é seu nome e sobrenome?	9. Você diria que seus cabelos são claros ou escuros?		
2. Por que você veio a esta consulta?	10. Você agora está sentado(a), de pé ou deitado(a)?		
3. Você tem algum problema de saúde que o(a) impede de	11. O que você planeja fazer hoje ou amanhã?		
levar uma vida normal?	12. Se você tivesse que viver sua vida novamente, recomeçando		
4. Você tem algum problema de memória?	tudo desde a infância, há algo que você gostaria de mudar?		
5. Você já teve algum trabalho ou emprego? Qual era sua	O que?		
ocupação?	13. Você acha que mentir é bom ou ruim? Por quê?		
6. Qual é o nome de seu marido (ou esposa)?	14. Você acha que dar comida ou dinheiro a alguém que está		
7. Qual é o nome de sua mãe?	passando fome é bom ou ruim? Por que?		
	
**Escala de Negação da Doença (Starkstein & Robinson, 1994)**			
**Perguntas**	**Pontuação**		
1. O paciente minimiza seus sintomas atuais durante a entrevista.	0 (não)	1 (1 ou 2 vezes)	2 (mais de 2 vezes)
2. O paciente diz não haver nada realmente errado com ele (ela) e	0 (não)	1 (1 ou 2 vezes)	2 (mais de 2 vezes)
que está pronto(a) para ir para casa.			
3. O paciente (no passado ou no presente) refere que a causa de seus	0 (não)	1 (1 ou 2 vezes)	2 (mais de 2 vezes)
sintomas está em outros órgãos que não o cérebro, ou queixa-se			
de sintomas não relacionados ao sistema nervoso central.			
4. Em algum momento o paciente admitiu temer a morte?	0 (sim)	1 (não)	
5. Em algum momento o paciente admitiu ter medo de ficar	0 (sim)	1 (não)	
inválido?			
6. O paciente nega verbalmente estar no hospital.	0 (de ne-	1 (às vezes)	2 (toda vez)
	nhum modo)		
7. O paciente demonstra, ao menos aparentemente, um modo	0 (não)	1 (1 ou 2 vezes)	2 (mais de 2 vezes)
descontraído, alegre e jovial de lidar com a vida.			
8. Durante a entrevista, o comportamento do paciente se	0 (não)	1 (1 ou 2 vezes)	2 (mais de 2 vezes)
caracteriza por indiferença, frieza e despreocupação.			
9. O paciente transfere o medo de sua própria doença para a	0 (não)	1 (ao menos 1 vez	
família e outros pacientes.		durante a entrevista)	
10. O paciente projeta sua doença ou fraqueza em seus familiares.	0 (não)	1 (ao menos 1 vez	
		durante a entrevista)	
